# The biology of cutaneous neurofibromas: Consensus recommendations for setting research priorities

**DOI:** 10.1212/WNL.0000000000005788

**Published:** 2018-07-10

**Authors:** Jean-Philippe Brosseau, Dominique C. Pichard, Eric H. Legius, Pierre Wolkenstein, Robert M. Lavker, Jaishri O. Blakeley, Vincent M. Riccardi, Sharad K. Verma, Isaac Brownell, Lu Q. Le

**Affiliations:** From the Department of Dermatology (J.P.B., L.Q.L.), UT Southwestern Medical Center, Dallas, TX; Dermatology Branch (D.C.P., I.B.), Center for Cancer Research, National Cancer Institutes of Health, Bethesda, MD; Human Genetics Department (E.H.L.), University of Leuven, Belgium; Division Cancer Immunity Transplantation Infections (P.W.), Paris Est Créteil University, France; Department of Dermatology (R.M.L.), Northwestern University, Chicago, IL; Department of Neurology (J.O.B., S.K.V.), The Neurofibromatosis Therapeutic Acceleration Program, The Johns Hopkins University School of Medicine, Baltimore, MD; and The NF Institute (V.M.R.), La Crescenta, CA.

## Abstract

**Objective:**

A group of experts in dermatology, genetics, neuroscience, and regenerative medicine collaborated to summarize current knowledge on the defined factors contributing to cutaneous neurofibroma (cNF) development and to provide consensus recommendations for future research priorities to gain an improved understanding of the biology of cNF.

**Methods:**

The group members reviewed published and unpublished data on cNF and related diseases via literature search, defined a set of key topic areas deemed critical in cNF pathogenesis, and developed recommendations in a series of consensus meetings.

**Results:**

Five specific topic areas were identified as being relevant to providing an enhanced understanding of the biology of cNF: (1) defining the human cells of origin; (2) understanding the role of the microenvironment, focusing on neurons, mast cells, and fibroblasts; (3) defining the genetic and molecular differences between the cNFs, focusing on size and number; (4) understanding if sex hormones are critical for cNF development or progression; and (5) identifying challenges in establishing in vitro and in vivo models representing human cNF.

**Conclusions:**

The complexity of cNF biology stems from its heterogeneity at multiple levels including genetic, spatial involvement, temporal development, and cellular composition. We propose a unified working model for cNF that builds a framework to address the key questions about cNF that, when answered, will provide the necessary understanding of cNF biology to allow meaningful development of therapies.

The clinical spectrum of neurofibromatosis type 1 (NF1) is broad and dependent on in which cell type the biallelic inactivation of the *NF1* (*Neurofibromin 1*) gene takes place, such as melanocytes (café-au-lait macules [CALM]), osteoblasts (tibial dysplasia), and Schwann cells (cutaneous neurofibroma [cNF] and plexiform neurofibroma [pNF]). Importantly, the 2 main types of neurofibroma (cNF and pNF) differ widely clinically. cNF typically become apparent around puberty and increase in number with age. They reside exclusively in the dermis and never progress to malignancy. In contrast, pNF are often congenital, and progress predominantly during childhood and adolescence. Although pNF can involve the skin, they are generally deep tumors involving nerve plexuses below the dermis and they carry a risk of sarcomatous transformation. Despite knowing that the NF1 syndrome and its manifestations are caused by mutation of the *NF1* tumor suppressor gene, there is little understanding about why the multiple manifestations of NF1 occur in some people but not others and with variable severity, even within families.

The most common tumor in adults with NF1 is cNF, presumably resulting from the biallelic loss of *NF1* in the Schwann cell lineage. cNF can vary widely in size (from millimeters up to few centimeters) and in numbers (from a few to thousands that can cover most of the skin surface) and can develop in virtually any location of the body. In addition to the important psychosocial consequence of cNF tumors, irritation, bleeding, pain, and superficial infections are unmet clinical needs. There are no preventative measures, and the only treatment options are surgical.

Researchers interested in understanding cNF biology face multiple critical challenges. First, the human *NF1* gene and its mRNA are relatively large^1^ and difficult to manipulate into expression vectors. Therefore, the biochemistry of the different protein domains of NF1 (except for the GTPase-activating protein [GAP] domain) is not well-understood.^2^ Second, cNF are slow-growing, noncancerous tumors. Consequently, isolating and maintaining primary cultures from cNF is difficult. Third, the generation of robust 3D in vitro culture systems^3^ and in vivo models^4^ are notoriously challenging. In an effort to address these gaps, based on existing data from both cNF and pNF literature, this group sought to identify the key gaps in the understanding of cNF biology that require investigation in order to accelerate the development of effective therapies for these tumors.

## Methods

A literature review was conducted including the terms NF1, cNF, dermal neurofibroma, pNF, genotype, phenotype, mast cell, macrophage, fibroblast, fibrosis, hormones, development, and animal models in an effort to identify all published literature that may be relevant to the general topics of cNF initiation, development, and progression. The working group was composed of experts in Schwann cell biology, genetics, dermatology, stem cell biology, fibrosis, regenerative medicine, and NF1 clinical care. Working group members reviewed published and unpublished data around the set of topic areas individually and as a group during a series of meetings facilitated over a 4-month period, prioritized key questions, and established consensus recommendations for each topic area.

## Results

### Defining the human cells of origin

Theoretically, the cell of origin is the cell that first undergoes *NF1* biallelic inactivation, regardless of the timing of this event. Identification and isolation of the human cells of origin would allow reconstruction of the biological steps leading to tumor initiation and progression ultimately leading to identification of targets that may be susceptible to therapeutic intervention.

**Figure 1:**
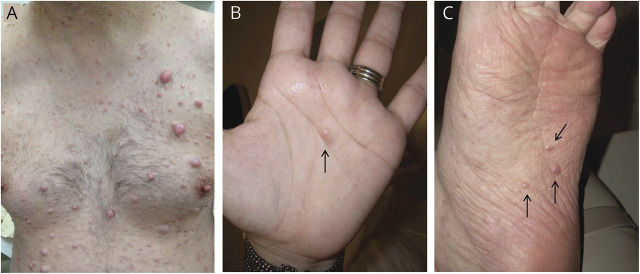
Clinical cutaneous neurofibroma examples across body regions Cutaneous neurofibromas can develop on hair-bearing skin (A) or non-hair-bearing skin on the palm (B) or sole (C) (arrows).

Several groups have reported a putative cell of origin for cNF. Karvonen et al.^5^ proposed that cNF originate from human hair follicle stem cell–like neurofibroma precursor cells. However, patients with NF1 also develop cNF on the palms and soles where there are no hair follicles (figure 1). Le et al.^4^ observed pNF when *Nf1*^*−/−*^ skin-derived precursor cells (SKPs), a population of neural crest–derived progenitors in the murine dermis, were implanted into the injured sciatic nerve of immune compromised mice. However, cNFs only developed when these cells were implanted in the skin of hormone-primed recipients or in a topically inducible model deleting *Nf1* locally in the skin.^4^ Of note, biallelic inactivation of *Nf1* in pNF models does not robustly lead to cNF, supporting a hypothesis that the cells of origin of cNF and pNF are different. Interestingly, Gresset et al.^6^ reported that the Schwann cells of the dermal nerve endings have a different origin compared to the majority of the other Schwann cells. Intriguingly, these neural crest–derived stem cells are reminiscent of the SKPs^4,7^ and suggest that they may be the embryonic source of the cNF cell of origin. In this sense, identifying mouse Cre lines that are expressed in the subpopulation of SKPs that give rise to cNF will be instrumental to our understanding of cNF biology. Other indirect evidence supporting the differential origin of cNF and pNF comes from experiments aiming to elucidate the contribution of skin or nerve trauma in the development of neurofibroma. Strikingly, cNF was never observed in an injured sciatic nerve–dependent pNF mouse model where deep skin incision was performed. This suggests that wounded small nerve endings do not behave like the sciatic nerve in their ability to promote neurofibroma. Directly assessing the effect of dermal nerve injury in existing^4^ or novel cNF mouse models will help clarify the role of injury in cNF formation. Importantly, the cell of origin of cNF may encompass melanocytes as skin hyperpigmentation completely covers some cNF (diffuse cNF). Some cNF present with redness or purplish hue, likely due to tumor vascularization (figure 1). Collectively, these data and observation indicate a need to better characterize the types of nerve associated with cNF and pNF regarding their intrinsic tumorigenic properties and determine if there is evidence supporting the hypothesis that cNF and pNF have distinct cellular origin.

### Understanding the tumor microenvironment

The cell types found in either human cNF or pNF are virtually the same as the cell types found in healthy peripheral nerves except they are mingled in a collagen-rich, fibrotic extracellular matrix (ECM) and are highly disorganized. Current data suggest that the most important cellular components for development and maintenance of cNF are the nerve, immune cells (e.g., mast cells), and fibroblasts.

**Figure 2:**
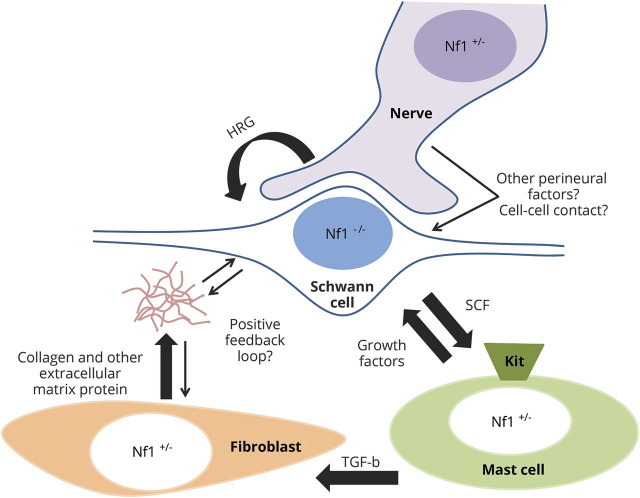
Paracrine signaling working model in neurofibroma Schwann cells, which depend on the nerve and heregulin (HRG) to proliferate, secrete stem cell factor (SCF). SCF binds to the receptor kit on mast cell, which in turn stimulates collagen deposition through activation of fibroblast by the transforming growth factor–β (TGF-β).

Normal Schwann cells require nerve contact during development.^8^ Adameyko et al.^9^ described the migration of Schwann cell precursors (SCPs) along growing nerves, and demonstrated that the association with nerves influences the fate of the precursor cells. In the context of neurofibroma, Liao et al.^3^ demonstrated that murine *Nf1*-deficient SKPs more readily give rise to pNF when injected into nerve tissue, but they did not develop pNF when injected into non-nerve tissue. Further work is needed to clarify if human cNF may also require contact with nerves or factors such as Neuregulin 1 (NRG1) in the perineurial microenvironment for their development and maintenance (figure 2).

The immune cells that contribute to cNF are mast cells and macrophages. Mast cells are histamine-secreting granulocytic cells known for their involvement in allergic reactions. Mast cell infiltration is one of the histologic hallmarks of neurofibroma.^10^ A working model of tumorigenesis derived from experiments conducted mainly using a mouse pNF model proposes Schwann cell–mast cell–fibroblast paracrine signaling (figure 2).^11^ Specifically, the Parada laboratory showed that the Krox20Cre *Nf1*^f/-^ pNF mice only developed pNF in a *Nf1*^*+/−*^ background, supporting the hypothesis that non-Schwann cell types are sensitive to *Nf1* gene dosage and may play an important role in pNF development.^12^ Subsequently, the Clapp laboratory showed that deletion of *Nf1* in Schwann cells induces a higher secretion of stem cell factor which in turn recruits *Nf1*^*+/−*^ mast cells through kit receptor activation.^13^ Hypermotile *Nf1*^*+/−*^ mast cells subsequently activate *Nf1*^+/−^ fibroblasts and stimulate their collagen deposition through tumor growth factor–β (TGF-β),^14^ explaining a second hallmark of human pNF: abundant collagen deposition.^11^ However, it is not clear to what extent these data can be extrapolated to human cNF. Clinical application of these findings via a Kit receptor inhibitor or mast cell stabilizer showed only modest results in clinic for pNF.^15,16^ One possibility is that the pNF mouse model is more dependent on the Schwann cell–mast cell axis than human pNF. Also, these models did not account for macrophages that are commonly found in cNF. Although infiltration of macrophages correlates with tumor progression, their exact role in neurofibroma tumorigenesis is not well-understood.^17^

Finally, fibroblasts are nonepithelial, noninflammatory, and nonvascular cells of the connective tissue responsible for ECM deposition and reorganization.^18^ Upon skin injury, the normal wound repair process includes inflammation followed by fibroblast proliferation, re-epithelization, and ECM remodeling. Fibrosis, defined as formation of excess connective tissue in an organ, occurs during the second phase of healing. High levels of collagen deposition, as well as the expression level of key components of the TGF-β pathway in activated fibroblasts, are surrogates often used to quantify the extent of fibrosis. No treatment is currently able to reverse fibrosis, but drugs inhibiting the TGF-β pathway are under investigation for this purpose.^19^

Neurofibroma-associated fibroblasts (NAFs) are abundant in cNF, where up to 50% of human cNF dry weight is collagen and NAFs display abnormal collagen deposition, all hallmarks reminiscent of fibrosis.^20^ However, initial attempts to verify the role of fibroblasts in human pNF by pharmacologically targeting fibroblast-producing collagen have had limited success.^21,22^ One explanation is that NAFs may not be the typical activated fibroblast cell characterized in major organ fibrosis and cancer contexts. Indeed, NAFs do not express fibroblast markers observed in other cutaneous disorders like dermatofibrosarcoma protuberans^23^ or keloid,^24^ and are negative for smooth muscle actin, a classic marker for activated fibroblasts.^25^ Alternatively, collagen-producing fibroblasts may not be functionally equivalent in cNF and pNF. Overall, multiple questions remain about the role of NAFs in cNF pathogenesis.

An interesting theory that bridges the hypotheses of fibrosis, neurofibroma formation, and a hyperactive immune response is that neurofibromas may develop in the setting of trauma.^26,27^ Indeed, nerve injury initially attracts immune cells such as macrophages (to clear cellular debris during Wallerian degeneration)^28^ followed by mast cells (likely to increase capillary permeability).^29^ Specifically in the context of the peripheral nerve system, proliferating fibroblasts and Schwann cells are aligned in an ordered column (also known as band of Bungner) to bridge the gap between the distal and proximal nerve, allowing guidance for axonal regrowth.^30^ Finally, the ECM is slowly remodeled to eventually reach normal architecture. In the context of neurofibroma, one can envision that the high number and proliferative status of Schwann cells triggered by nerve injury and loss of axonal contact would significantly increase the likelihood of biallelic *NF1* inactivation. How the ECM produced by fibroblasts and the recruited immune cells initially aiming at healing the nerve would then turn into a fibrotic microenvironment preventing the nerve to heal is currently unknown. What is increasingly clear is that for the best chance of effective therapeutics, prior to investigating treatments targeting various elements of the cNF microenvironment, a better understanding of the relative contributions of each of these various cells to tumorigenesis is needed. This will entail (1) identifying specific markers for each cell type described and their relative number and function in various phenotypes of cNFs^31^ and (2) the development of reliable in vitro and in vivo model systems that recapitulate human cNF progression to allow omission of specific cell types and enable pharmacologic/genetic interventions.^6^

Other cell types in the microenvironment of cNF include pericytes, and cellular components comprising the nerve perineurial barrier, but their role is unclear.^32^ Of note, keratinocytes, melanocytes, and other cells from the skin structures (e.g., sebaceous gland, hair follicle, and eccrine gland) can be found at the margin of cNF but not within the tumor bulk. Finally, it is possible, but uncommon, to find abundant adipocytes or fat-like cells in neurofibromas.^33^

### Defining the genetic and molecular differences between the cNFs: Focus on size and number

There is extensive interpatient and intrapatient heterogeneity in the presentation, degree of severity, and behavior over time of cNF. Efforts at defining genotype–phenotype correlations in NF1 in general and relative to cNF have largely been fruitless, with 3 notable exceptions. The first 2 exceptions are specific *NF1* gene mutations that result in a very low incidence of cNF or pNF (c.2970-2972delAAT; p.992delM^34^ and R1809 *NF1* mutations^35^) and the third results in a higher likelihood of early development and higher number of cNF (large *NF1* gene deletions).^36^ It would be highly informative to validate the biochemical and biological consequences of mutated neurofibromin to better understand how these first 2 mutations “suppress” cNF development. Specifically, how do these changes alter 3D protein structure, influence sites for binding partners, or affect subcellular localization of neurofibromin? Even more intriguing is that this mild cutaneous phenotype is indistinguishable from Legius syndrome, which is caused by mutations in the Ras signaling regulator *SPRED1,*^37,38^ suggesting the major influence of this mutation is via the modulation of the Ras signaling pathway in melanocytes and other cells, but not significantly in Schwann cells. Further, these 2 mutations do not directly implicate the GAP domain of neurofibromin, so it is unclear how mutations at R1809 or c.2970-2972delAAT would affect the Ras signaling inhibitory capacity of neurofibromin. Elucidating the mechanism behind the lack of development of cNF in patients with mutations in *NF1* R1809 or *NF1* c.2970-2972delAAT could be groundbreaking for new therapeutic approaches for cNF.

In sharp contrast, around 5%–10% of NF1 patients have a large deletion encompassing the *NF1* gene, which is associated with a high number of cNF and an increased risk for malignant peripheral nerve sheath tumor (MPNST). The genetically linked *SUZ12* gene has been identified as an important modifier of NF1 in MPNST.^39^ It is unknown if *SUZ12* or any other gene that is commonly inactivated with NF1 in the setting of these large gene deletions plays a role in determining the number, size, or timing of onset of cNF.

Outside of these examples, for the majority of *NF1* DNA mutations, the predicted functional consequence at the RNA and protein levels is unknown, contributing to the lack of genotype–phenotype correlation. By systematically annotating the RNA and protein effects due to different *NF1* mutations in tissues and cells from NF1 patients,^40–42^ one can begin grouping together apparently unrelated mutations.^43,44^ An even better approach is systematic functional profiling of mutant neurofibromin. Scoring the effect on Ras signaling may help reclassify discordant results, generating new hypotheses about the role of neurofibromin outside its GAP domain and new mechanistic insights for cNF development.

There is often wide variation of cNF tumor size and concentration on patients' bodies, suggesting that either tumor growth is under the influence of unidentified stochastic modifiers or not all somatic mutations (the second hit mutation) leading to biallelic loss of *NF1* are equivalent. In support of the latter hypothesis, the mutational landscape of cNF across 40 tumors from 11 patients revealed that the nature of the second hit mutation in *NF1* is different in each tumor.^45^ It is currently unknown if there are different forms of cNF or if there are relatively few types of cNF that appear phenotypically distinct because they are observed at various stages along a growth continuum.^31,46^ A challenge is that cNF may not be visible at early stages and some patients report that cNF erupt over very short intervals. Hence, researchers investigating factors that explain the heterogeneity of cNF should be mindful that we do not yet have an adequate understanding of the natural history of cNF in humans.

### Understanding if sex hormones are critical for cNF development or progression

There have been many proposed factors that influence the development and growth of cNF.^31,46^ One of the more pervasive hypotheses is that pregnancy or other periods of systemic hormonal shifts stimulate cNF appearance and growth. Dugoff and Sujansky^47^ retrospectively surveyed 105 women with NF1 who had a history of at least one pregnancy to assess perceived change in number or size of cNFs during prior pregnancies. More than half (64/105) of the women reported that new cNFs developed during pregnancy, but multiparous women did not have new tumors develop during all pregnancies. Lammert et al.^48^ distributed a survey to 59 women with NF1 who had used hormone contraceptives to evaluate the effect of exogenous estrogen and progesterone on the development or proliferation of cNFs while on these drugs. The majority (53/58) of women reported no change in cNFs, but 5 reported new cNFs and growth of existing ones. Although interesting, these studies are inconclusive as they suffer from their retrospective and subjective nature. Notably, Schwann cells derived from human neurofibromas express progesterone receptors, and they have elevated proliferation rates when exposed to progesterone in vitro*.*^49^ Further, Le et al.^4^ showed that implantation of *Nf1*^−/−^ SKPs resulted in development of cNFs only in pregnant *CMV-Cre*^*ERT2*^*;Nf1*^*f/−*^ mice, adding preclinical data to the working hypothesis of an association between sex hormone exposure and cNF development in female patients. However, Sbidian et al.^50^ analyzed prospective data collected between 2002 and 2005 from adults (mean age 36 ± 14 years) with NF1 to assess difference in growth and number of cNF between sexes and between nulliparous and parous women. Men were at higher risk of developing subcutaneous neurofibromas regardless of age, but there was no difference in the number of cNFs between men and women after the age of 40, regardless of the parous status of the women. Overall these data indicate that there may be a role of sex hormones in development of cNF; however, they are not causative or even dominant. Prospective epidemiologic case–control studies that address different phases of hormonal changes in women with concurrent assays of systemic hormonal levels (i.e., during exogenous hormone exposure, pregnancy, and in menopause) are important for understanding of the role of hormones at different phases of cNF development clinically.

### Challenges in establishing in vitro and in vivo models representing human cNF

Developing cNF models will be critical to further elucidate their biology and to serve as preclinical models for therapeutic testing. However, there are many challenges in establishing in vitro and in vivo models of human cNF. First, genetically, cNF initiation and progression must associate with *NF1* loss. Tumor development should be driven by specific changes in known pathways involved in human cNF, and tumor gene expression profiles should resemble those of human cNF. Second, biologically, cNFs develop through multistage progression (initiation, proliferation/progression, and quiescence). The cNF models should initiate from a small group of cells of origin that harbor *NF1* loss, and the tumor histology and pathology should be similar to human cNF. In addition, tumor development should require similar microenvironmental factors as in human cNF. Third, therapeutically, new cNF models should be robust for the preclinical testing of inhibitors directed at pathways critical for cNF development that can lead to clinical trials in humans. To better understand the role of the tumor microenvironment on the development and proliferation of cNF, future studies should identify what extrinsic signals regulate cNF formation, which includes understanding the cell source of these signals, the timing of these signals, if biallelic inactivation in the cells is important, and elucidating the role of soluble signaling molecules, ECM, and cell contact.

## Discussion

Analysis of NF1 patient tumors and mouse models has led to the conclusion that the loss of function of *NF1/Nf1* is an early and necessary step in the development of neurofibromas. However, we do not know what further triggers or modifies neurofibroma growth to yield the wide range of intrapatient and interpatient variability that is seen. Assuming different cellular origins for the different phenotypic manifestations of the disease, it is possible that the different thresholds for functional *NF1* from one cell type to another explain the variable phenotypes observed: melanocytes (CALMS, Lisch nodules) < cNF cell of origin (cNF) < Schwann cell lineage (pNF). This hypothesis would explain why even mildly affected patients with NF1 have CALMS, why patients with severe pNF almost always carry CALMS and cNF, and why the phenotypic manifestations have different temporal onset. Each *NF1* DNA mutation potentially has more or less consequence because it leaves neurofibromin with more or less residual function and some cell types are more dependent on NF1 function than others. Importantly, this theory does not exclude a role for the tumor microenvironment and more study is needed here.

As we progress in our quest to decipher the biology of cNF, it remains important to compare and contrast cNF and pNF in terms of cellular origin, *NF1* genotype and modifiers, cellular composition, and the effect of tumor microenvironment, as well as the effects of sex hormones. A top priority is to continue to gather large repositories of *NF1*-driven neurofibromas with annotated clinical data so that correlations can be delineated. Finally, analysis of these biospecimens should be expanded by including transcriptomic, proteomic, and epigenetic profiling through unified collaborative approaches to accelerate discovery most efficiently.

## Glossary

CALM: café-au-lait macule
cNF: cutaneous neurofibroma
ECM: extracellular matrix
GAP: GTPase-activating protein
MPNST: malignant peripheral nerve sheath tumor
NAF: neurofibroma-associated fibroblasts
NF1: neurofibromatosis type 1
pNF: plexiform neurofibroma
SKP: skin-derived precursor cell
TGF-β: tumor growth factor–β
